# Analysis of implementation outcomes of quality improvement initiatives in Haiti: the fingerprint initiative

**DOI:** 10.26633/RPSP.2021.68

**Published:** 2021-05-26

**Authors:** Joseph Adrien Emmanuel Demes, Victor Becerril-Montekio, Pilar Torres-Pereda, Ernst Robert Jasmin, Jean Geto Dube, Jean Garcia Coq, Nathan Nickerson

**Affiliations:** 1 State University of Haiti Port-au-Prince Haiti State University of Haiti, Port-au-Prince, Haiti; 2 Instituto Nacional de Salud Pública Cuernavaca Mexico Instituto Nacional de Salud Pública, Cuernavaca, Mexico; 3 Ministry of Public Health and Population Cap-Haitien Haiti Ministry of Public Health and Population, Cap-Haitien, Haiti; 4 Konbit Santé Cap-Haitien Haiti Konbit Santé, Cap-Haitien, Haiti

**Keywords:** Quality improvement, implementation science, biometric identification, Haiti, Mejoramiento de la calidad, ciencia de la implementación, identificación biométrica, Haití, Melhoria de qualidade, ciência da implementação, identificação biométrica, Haiti

## Abstract

**Objective.:**

To assess the process and outcomes of the implementation of an electronic fingerprint initiative as part of quality improvement in three health facilities in the Northern Department of Haiti, in terms of its acceptability, adoption, feasibility, fidelity, and sustainability. In Haiti, poor attendance of the healthcare workforce is a nationwide problem, closely related to the quality of care. Three health institutions have tried to implement an electronic fingerprint system to monitor and improve attendance.

**Methods.:**

An exploratory and qualitative descriptive study of the implementation outcomes of the fingerprint initiative. It was based on semi-structured interviews and one group discussion using purposeful sampling techniques to recruit participants, and an open coding system and deductive approach to analyze the data using ATLAS.ti 8.

**Results.:**

The fingerprint initiative was successfully implemented in a non-governmental organization supported health facility but, despite some planning, it was never implemented in the public health facilities. The acceptability of the implementation was high in the not-for-profit organization and low in the public settings, mostly in relation to the presence of champions and the leadership at each health facility.

**Conclusions.:**

We recommend more involvement of the leadership of health facilities in the different phases of the implementation process in order to guarantee acceptability, adoption, fidelity and sustainabiliy. More research is needed to articulate this technology-driven initiative in the Haitian health system.

## RESUMEN

**Objetivo.:**

Evaluar el proceso y los resultados de la puesta en práctica de una iniciativa de digitalización de huellas dactilares como parte de la mejora de la calidad en tres establecimientos de salud del departamento Norte de Haití, en cuanto a su aceptabilidad, adopción, viabilidad, fidelidad y sostenibilidad. En Haití, el absentismo de los trabajadores de atención de salud es un problema nacional, estrechamente relacionado con la calidad de la atención. Tres instituciones de salud han tratado de poner en práctica un sistema de huellas dactilares digitalizadas para monitorear y mejorar la asistencia.

**Métodos.:**

Se ha llevado a cabo un estudio descriptivo cualitativo y exploratorio de los resultados de la puesta en práctica de la iniciativa de la huella dactilar. Este estudio empleó entrevistas semiestructuradas y una discusión de grupo mediante técnicas de muestreo con fines específicos para reclutar a los participantes, y un sistema de codificación abierta y enfoque deductivo para analizar los datos con ATLAS.ti 8.

**Resultados.:**

La iniciativa de la huella dactilar se llevó a cabo satisfactoriamente en un establecimiento de salud no gubernamental; sin embargo, a pesar de cierta planificación, no llegó a ponerse en práctica en establecimientos públicos de salud. La aceptabilidad de la implementación fue alta en las organizaciones sin fines de lucro y baja en los entornos públicos, principalmente en lo que respecta a la presencia de líderes y dirigentes en cada establecimiento de salud.

**Conclusiones.:**

Recomendamos una mayor participación de la gerencia de los establecimientos de salud en las diferentes fases del proceso de ejecución para garantizar aceptabilidad, adopción, fidelidad y sostenibilidad. Se necesita investigación adicional para integrar esta iniciativa impulsada por la tecnología en el sistema de salud haitiano.

## RESUMO

**Objetivo.:**

Avaliar o processo e os resultados da implantação de uma iniciativa de controle de ponto eletrônico por biometria para melhoria da qualidade em três serviços de saúde no Departamento Norte do Haiti quanto à aceitabilidade, adoção, viabilidade, fidelidade e sustentabilidade. A baixa assiduidade no trabalho do pessoal da saúde é um problema em todo o país e está estreitamente relacionada à qualidade da atenção. Foi feita uma tentativa de implantar um sistema de ponto eletrônico por biometria em três instituições de saúde, para monitorar e aumentar a assiduidade no trabalho.

**Métodos.:**

Estudo exploratório, descritivo e qualitativo dos resultados da implantação da iniciativa de ponto eletrônico por biometria. Os dados foram coletados em entrevistas semiestruturadas e em um grupo de discussão usando técnica de amostragem intencional para recrutar os participantes. A análise de dados foi realizada por codificação aberta e enfoque dedutivo com o uso do software ATLAS.ti 8.

**Resultados.:**

A iniciativa de ponto eletrônico por biometria foi implantada com êxito em um serviço de saúde apoiado por uma organização não governamental. Porém, apesar do planejamento, ela nunca foi implantada nos serviços da rede pública. A aceitabilidade foi muito boa na entidade sem fins lucrativos, mas foi baixa na rede pública, sobretudo em relação à presença de apoiadores e dirigentes no local.

**Conclusões.:**

Recomenda-se maior envolvimento dos dirigentes dos serviços de saúde nas diferentes fases do processo de implantação para garantir a aceitabilidade, adoção, fidelidade e sustentabilidade da iniciativa. Mais pesquisas são necessárias para estruturar esta iniciativa com uso de tecnologia no sistema de saúde do Haiti.

Today, health authorities have enough international evidence on what needs to be done to improve health systems’ performance. Relevant questions are about how to uptake proven interventions and implement them (1). Among the main issues that health systems are still concerned about is the quality of care.

Quality improvement strategies can be defined as a set of coordinated activities to improve the process of care and patient satisfaction (2). Quality improvement initiatives (QIIs) involve a set of structured and cyclical processes, often called the plan–do–study–act cycle, and use scientific methods on a continuous basis to formulate a plan, implement the plan, and analyze and interpret the results. The introduction of change in order to improve the quality of care is always an iterative process.

In Haiti, QIIs have been widely implemented, beginning with the HEALTHQUAL-Haiti program in 2007. This paper focuses on one of four QIIs that were a part of a single study conducted during 2019 with the support of the Pan American Health Organization (PAHO) and the Alliance for Health Policy and Systems Research. Its general aim was to understand how these quality-oriented interventions were being implemented with a view to improving their performance, assuming that implementation research is essential to understand how they had been planned and executed.

Implementation research helps decisionmakers, policymakers, and practitioners to successfully implement evidenced-based health interventions (1). It considers the factors affecting implementation, its processes, and its results, including how to introduce potential solutions or how to promote their large-scale use and sustainability. Its purpose is to understand what, why, and how interventions work in real-world settings and to test approaches to improve them (3).

Good human resources management is a key element to improving quality of care. Several studies in low- and middle-income countries have highlighted the problem of absenteeism in clinical settings and its adverse impact on patient care (4). Absence rates have been found to be higher in the poor regions (5), ranging from 25% to 70% (6–9).

To better monitor the presence of staff, several health facilities have tried to introduce a biometrics-based attendance system that uses the fingerprint of employees to document who is punching in and out of work. This system has several advantages over paper systems (10), such as saving time (11). Employees do not need to perform any physical task other than touching the fingerprint scanner to sign in or out. Each individual has a unique fingerprint that cannot be duplicated, minimizing the possibility of other staff signing in for a coworker, making it a more reliable proof of employees’ contribution in terms of time dedicated to the work (11). The system can be programmed to automatically generate synthesis reports (12).

Since absenteeism has serious consequences in terms of mortality, morbidity, and the quality of care (9, 13), this technology is of great interest to the Ministry of Health Directorate in the Northern Department of Haiti, with a view to improving human resources management and accountability. Its implementation, however, has been challenging.

This study aims to explore the QIIs in the context of Haiti by assessing the process and outcomes of the implementation of the fingerprint initiative in three health facilities in the Northern Department. Building on the implementation outcome variables (3), this study focuses on the acceptability, adoption, feasibility, fidelity, and sustainability of the implementation of the fingerprint initiative as part of the quality improvement programs.

## MATERIALS AND METHODS

This is an exploratory and qualitative descriptive study based on the implementation research conceptual framework to describe stakeholders’ perceptions of a quality improvement intervention in the field. This approach aims to produce a straight description and comprehensive summary of the phenomenon using participants’ language and staying close to the data (14).

Three health centers were selected to analyze the implementation process and outcomes of one QII based on Peters’ proposal regarding the dimensions of analysis in implementation research (3).

### Conceptual framework and research design

This study uses the implementation research framework, defined as the scientific investigation of aspects related to how interventions are implemented and perform in specific contexts. It focuses primarily on the study of factors that influence the execution of interventions, the implementation process itself, and its outcomes (3, 15). It focused specifically on the acceptability, adoption, feasibility, fidelity, and sustainability of the implementation of the fingerprint system used to monitor attendance and punctuality at the health facilities (Table 1).

An exploratory design was chosen to look at possible factors affecting the implementation and to shed light on vital aspects of the implementation results in three health facilities in the Northern Department of Haiti from April to October 2019.

### Sampling

We used purposive sampling to select research sites and participants (16). The health facilities were selected considering the important information they can provide that cannot be obtained elsewhere (17); because they were selected by the Ministry of Health (MOH) to implement this new QII, and because they have two organizational schemes: one mix (MOH and non-governmental organization (NGO)), managed by an NGO or not-for-profit organization; and two public units, under the responsibility of the MOH.

### Data collection

Data were collected through semi-structured interviews (*n* = 20) and one group discussion (*n* = 4) because it was difficult to meet these people individually. One interview was conducted by phone. The interview guides contemplated questions related to the implementation of the fingerprint initiative, and they were revised as they accommodated additional emerging themes. Most interviews were conducted in Haitian Creole or French by trained qualitative researchers. One interview was conducted in English. They were audio-recorded and transcribed for analysis, except for one semi-structured interview and the small group discussion, as some participants were not comfortable with the recording. Data were transcribed into a Word document. Written informed consent was obtained from the respondents prior to each interview.

**TABLE 1.** Dimensions to assess the fingerprint initiatives in three health facilities, Northern Department, Haiti, 2019

Dimension	Definition
Acceptability	The perception among stakeholders (e.g., consumers, providers, managers, policymakers) that an intervention is agreeable.
Adoption	The intention, initial decision, or action to try to employ a new intervention.
Feasibility	The extent to which an intervention can be carried out in a particular setting. It looks at the practicality, actual fit, utility, trialability setting or organization.
Fidelity	The degree to which an intervention was implemented as it was designed in an original protocol, plan, or policy.
Sustainability	The extent to which an intervention is maintained or institutionalized. Maintenance, continuation, durability, institutionalization, in a given setting routinization, integration, incorporation.

***Source:*** Peters et al., 2013 (3).

### Data analysis

Acceptability, adoption, feasibility, fidelity, and sustainability were selected as the focus of our analysis using an inductive and deductive approach (18) and ATLAS.ti 8 (19, 20). The codebook was tested deductively to assess the outcomes of implementation. We also analyzed the data inductively in order to judge and find agreements to support each domain in the outcomes of implementation and assess the degree of implementation in relation to the implementation outcome variables in the setting under study.

We used constant comparative methods in order to compare data to data, codes to codes and codes to data, and categories to data and categories to categories (21, 22). We elaborated memos during the analysis process in order to build a step-by-step understanding of the process (21). The data were analyzed by three people, and an intercoder reliability exercise was performed.

The final interpretation of the findings, as presented in this manuscript, emerged through active discussion among the co-authors.

### Ethics

The rights of the participants were respected, and informed consent was obtained from them. All identifying data were codified to guarantee participants’ anonymity. The study was approved by the Haiti National Bioethics Committee and the PAHO Ethics Review Committee.

## RESULTS

We present findings according to the five categories at the core of this study. Table 2 presents the key demographic data about the participants. Table 3 presents the main implementation outcomes.

**TABLE 2.** Demographic data, health facilities and NGO, Northern Department, Haiti, 2019

Characteristics	Health facilities			NGO
	HF1	HF2	HF3	NGO personnel
**Management of health facility**	NGO	Public	Public	NA
**Total participants**	5	10	7	2
Interviews	5	10	3	2
Group discussion	0	0	4	0
**Sex of participants**				
Female	3	6	4	1
Male	2	4	3	1
**Profession of participants**				
Medical doctor	1	2	6	0
Nurse	2	7	1	2
Administrator	2	1	--	--
**Position in the health facility**				
Management	1	2	0	1
Administration	2	1	0	0
Doctor, patient care	0	1	6	0
Nurse, medical care	2	6	1	0
Other	0	0	0	1

HF, health facility; NA, not applicable

***Source:*** Prepared by the authors from the study data.

**TABLE 3.** Main results for each implementation outcome variable and health facility, Northern Department, Haiti, 2019

Implementation outcomes	Health facilities		
	HF1	HF2	HF3
**1. Acceptability**	The initiative was well accepted at this health facility. The leadership of the health facility actively promoted it among employees and key stakeholders to get their buy-in. Key stakeholders and employees found it fully acceptable.	Some employees had a negative perception of the initiative. They considered it a way for the administration to control the staff.	The leadership of this health facility (director) accepted the initiative. Yet, we found no data showing that it was acceptable for the staff.
**2. Adoption**	The leadership of the health facility officially adopted the fingerprint initiative. They developed communication and marketing to actively promote it among the staff.	The intention to use the fingerprint devices existed at the administration level, but other key stakeholders considered it an external project imposed on the institution. Influential and powerful actors did not fully adopt the initiative.	Officially, this health facility never adopted the fingerprint initiative.
**3. Feasibility**	The feasibility was high at this health facility. It was practical, with a fit between the perception and the organizational aspect. It was considered useful to improve performance because the fingerprint was incorporated as an indicator for the payroll.	Low feasibility. Most staff had a negative perception of the initiative due to a culture of absenteeism and impunity. No communication plan to explain the system to the employees.	It was feasible but the initiative was never implemented.
**4. Fidelity**	The intervention was fully implemented as planned. The staff own the whole process. It was integrated in the organizational fabric of the health facility.	The initiative was never implemented despite the planning process and the availability of the materials.	The initiative was not implemented.
**5. Sustainability**	Highly likely to be institutionalized, as the initiative is owned by the staff and the leadership of the health facility.	Unlikely to be institutionalized.	Unlikely to be institutionalized.

HF, health facility

***Source:*** Prepared by the authors from the study data.

The final research sample consisted of 24 individuals (10 men and 14 women), purposely selected in the three health facilities and related NGOs. All participants were involved in QII implementation and were knowledgeable about the subject. Two were representatives of NGOs working outside the health facilities who, eventually, also work providing care at the public health facilities, while their pay comes from the NGO. The other 22 were administrators, management leaders of health facilities, and providers. All of them were key actors who would bring a wealth of findings to the study (Table 2).

This section provides evidence on the key implementation outcomes findings.

### 1. Acceptability

Most participants at the NGO-supported health facility developed a good perception of the fingerprint initiative, considering it fosters a collective form of leadership.

*(…) it is a collective leadership; the heads of services are co-leaders (…) It is with this kind of philosophy that we are still alive (…)* (HF1, Administrator 3).

*(…) each person is a leader in his field and exercises his leadership in his* field (HF1, Administrator 2).

In the public health facilities, instead, most of the personnel had a less favorable perception.

*It was a disaster* [the fingerprint] *because people thought it’s like we’re going to implement something to monitor us and to punish us later* (HF1, Medical Doctor 2).

*Because the problem is that they saw it as an external project that we wanted to impose on them (…)* (HF2, Medical Doctor 1).

The negative perceptions were fueled by the feeling that in the public system a certain degree of absenteeism is considered normal or acceptable.

*At the central level, there is a certain level of absenteeism that can be acceptable (…) at the peripheral level (…) there is* [also] *a level of absenteeism that is acceptable (…)* (HF2, Management 4).

*(…) There is a tendency in public health facilities that absenteeism could be normal (…) in the government system, people expect that you could be absent (…) people expect senators to be absent and not doing their jobs (…)* (HF2, Medical Doctor 1).

Some participants in the public health facility support the fingerprint as a tool to make the result-based financing system more transparent.

*I think that the fingerprint system could make it easier to monitor the attendance within the Result-Based Financing strategy (…) the goal is to assess the real contribution of staff (…)* (HF2, Nurse 1).

### 2. Adoption

The leadership involvement in the NGO-supported health facility facilitated the adoption and implementation of the fingerprint initiative.

*(…) The direction decides to use that system for the payroll as well (…) And people believed in it and got to work on time. The fingerprint system acted psychologically on people (…)* (HF1, Administrator 2).

They also developed a good communication system.

*(…) To have the confidence of all the staff, we needed a good communication so that all the staff could adopt it without forcing (…), and we had planned a meeting with all the staff (…) all the people (…) that were available were present (…) then I explained to them what we wanted to do (…)* (HF1, Nurse 1).

In the public health facility, the lack of communication was problematic.

*Well, I think that the major factor was a lack of communication (…) as any product (…) you must know how to conduct a kind of marketing; you must know how to sell the product to the buyer or the user (…) and it’s the hospital staff.* (HF2, Medical Doctor 1).

### 3. Feasibility

In the NGO health facility, the strategy is feasible as resources, both human and operational assets, were mobilized and the system prepared.

*(…) it is mandatory to have someone involved as a leader, not only must the leadership team of the institution get involved but there must be someone who must know that it is his responsibility to ensure that system is running smoothly (…) but without the support of leaders it will not work (…)* (HF1, Management 1).

*(…) The direction decides to use that system for the payroll as well (…) And people believed in it and got to work on time. The fingerprint system acted psychologically on people (…)* (HF1, Administrator 2).

Thus, the fingerprint was integrated in a more global strategy in order to be feasible and successful.

In the public health facility, even though the resources were available for the implementation, it was hard to do because the public health facility is a complex system presenting political, cultural, and structural barriers to any such initiatives.

*It’s not the same context because (…) it’s a private institution, there are safeguards, there are standards that are stricter (…) The staff has different orientations, different ideas (…) and different approaches (…) Public institutions have another philosophy rightly or wrongly but that’s the reality (…) We cannot replicate their model. (…) we can take models of public institutions but if we take the model of a private institution and we came to implement it in a public institution (…) it is a recipe for failure* (HF 2, Medical Doctor 1).

### 4. Fidelity

The fidelity to the original planning was high in the NGO-supported health facility and no implementation occurred at the public health facilities. In the public setting, the non-involvement of the leadership of the health facilities and their absence of interest in the implementation of the fingerprint system seems to be one of the principal barriers.

*If the leadership of (…) wanted it; the fingerprint system was going to work (…)”* (NGO, Staff).

*“Honestly, if the leadership does not want this to happen, it will not happen. (…) the leadership has its own reason why they* [public health facilities] *do not want it. The Departmental Health Direction can try to put pressure but if the leadership of the institution does not want it, it will not work.* (NGO, Management).

Besides, other structural, political, and cultural obstacles prevent the initiative from being implemented in the public health facilities.

*You do not think there is certain person if you install it* [the fingerprint] *(…) they will enjoy being irregular at work (…) just to see what could happen to them (…) because there are some of them who are employed by order of a Senator or a Member of Parliament (…) So the fingerprint will stress very deep things or anomalies in the system (…)* (HF2, Management 3).

*One of the obstacles in public institutions is the super employees who have more authority than the director sometimes (…) it is difficult to put this* [fingerprint] *system in place* (HF2, Management 4).

The same perceptions about the barriers experienced in the sites managed by the MOH were expressed by actors belonging to the health facility and managed by the NGO.

*Unfortunately, there was an undisclosed conflict between the administration and a staff member who is very influential (…) and he could cause others to no longer take care of the administration (…). And this caused division among the members (…) They should not divide but help each other, collaborate to manage the institution together.* (NGO, Staff).

### 5. Sustainability

In the NGO-supported health facility, the fingerprint became the norm and was integrated in the organizational fabric, creating conditions for sustainability.

*Absolutely, because it has become a habit (…) since the staff do not want to have absences or consecutively being late at the verification by the administrator (…) it changed their way of behaving and over time it is not a thing the administration has to force on or push for, but it became a habit, a normal behavior to be on time (…)* (HF1, Administrator 2).

Table 3 presents a summary of the main results in each health facility.

## DISCUSSION

These results shed light on the main obstacles to improve staff attendance and the quality of care with the implementation of fingerprint mechanisms in Haiti. To succeed, planning and implementation should consider these lessons. Acceptability and adoption of the implementation by stakeholders are key for a successful implementation of an innovation. However, the level of readiness of an institution toward an intervention is key for the implementation. Weiner (23) stresses the importance of organizational readiness for change and its impact on the success of the implementation. This readiness for change is influenced by the valence for change and the change efficacy. When the participants feel that they are not confident or have a negative perception of the innovation, it is less likely they will accept, adopt, and implement it (23).

Our results also describe the experience of other innovative initiatives to improve health care quality in Haiti where leadership and effective communication with health personnel play a central role. A key finding is that the implementation of the fingerprint initiative was more problematic in the public setting while it was successful in the NGO setting.

In the NGO setting, the intervention was implemented with fidelity to the original plan. This could be part of the debate about the fidelity of any implementation and the factors that influence it. Carroll and collaborators (24) develop a conceptual model that can be useful to help understand some of the key variables related to this.

Maybe the main difference between the two types of establishment (public and mix) is the capacity of the administration to impose sanctions on those who work in the establishment. In the NGO establishment there is talk of leadership, but behind it we recognize the capacity of the management to act on the personnel’s paycheck at the end of the month. Whereas in the public sector, there is a culture of impunity derived from habits of corruption and influence-peddling that leads to a lack of power. When there is no leadership, there is no accountability, and no one takes their responsibility to implement changes. So, the lack of leadership also explains the absence of implementation.

Our findings show that leadership is a key variable that influences acceptability, adoption, fidelity, and sustainability. When leaders are involved and own the process, a successful implementation is highly likely (2). Developing accountability systems and decentralizing human resources management so that health facilities can take effective disciplinary actions when staff are chronically absent could strengthen leadership. Besides, having a champion with key qualities to facilitate the process could be a main factor for the success of the implementation (25).

In this model there are potential moderators between the intervention and the adherence: participants’ responsiveness, comprehensiveness of policy description, strategy to facilitate implementation, quality of delivery, and the context (24). The NGO-supported health facility was more responsive and developed strategies and leadership for the implementation.

An intervention requires the interaction of different stakeholders at different levels. These actors can value the various aspects of the intervention differently. The implementation is a process and not a one-time event; it involves different stages or steps that are crucial for the success of the implementation (26, 27).

Furthermore, theory advancement is also a key challenge in implementation research. We need to open the black box to understand the process of implementation that leads to the results. Sometimes, people just look at the outcome without asking themselves how, or even if, the intervention has been implemented. Most of the time we observe a decision failure. People know what to do, but they do not decide to do it. In situations where people decide to implement and do not do it correctly, we could talk about an implementation failure (28). In the public system in Haiti, decision failure occurs often, despite all the planning.

Last but not the least, we noticed it was also a methodological challenge to measure the implementation outcomes using only a qualitative methodology. This is consistent with the literature on this topic. In the scientific literature, we also find several strategies to assess implementation outcomes with a focus on a quantitative approach (29). Michie and collaborators (29) notice that the main deficiencies concern the psychometric properties of the tools.

One of the main limitations of the study is that it was mainly based on the perceptions of the people at the health facilities and the NGO. The triangulation of the interviews with observation could have added more robustness to the research. Also, our sample was limited to three institutions in only one city where the strategy is being implemented; so, even for the context of Haiti, its results are not conclusive and its nature is exploratory rather than confirmatory. In the future, we could use mixed-methods approaches that would permit triangulation of the data. A more longitudinal study could be also necessary.

### Conclusion

These findings are relevant and new in the field of implementation research and shed light on specific aspects regarding the acceptability, adoption, fidelity, and sustainability of QIIs, particularly the fingerprint initiative. They also demonstrate that the perception of the staff is important in relation to the acceptability, adoption, and implementation of QIIs.

In the two public health facilities, most staff do not comply with the fingerprint initiative or have a negative perception about it as a strategy to improve the quality of care. As the experience of the NGO-managed health facility demonstrates, leadership is a key facilitator to the adoption and implementation of QIIs. Conversely, it is unlikely that the fingerprint system could be sustainable in the public health facilities without the involvement of their leadership. Based on these results, we recommend working on involvement of leadership and advocacy to improve the governance structure in order to facilitate the adoption, implementation, and sustainability of the fingerprint initiative in public health facilities. Using the fingerprint to create punctuality incentives instead of punishment mechanisms could also help change the stakeholders’ perceptions.

More research is needed to analyze and understand the outcome of implementation of QIIs, particularly in the Northern Department of Haiti. Future research should assess the acceptability, adoption, fidelity, and sustainability of the implementation of these initiatives in a prospective manner.

## Disclaimer.

Authors hold sole responsibility for the views expressed in the manuscript, which may not necessarily reflect the opinion or policy of the *RPSP/PAJPH* and/or PAHO.
